# The regulation of miRNAs by reconstituted high-density lipoproteins in diabetes-impaired angiogenesis

**DOI:** 10.1038/s41598-018-32016-x

**Published:** 2018-09-11

**Authors:** Samuel T. Hourigan, Emma L. Solly, Victoria A. Nankivell, Anisyah Ridiandries, Benjamin M. Weimann, Rodney Henriquez, Edward R. Tepper, Jennifer Q. J. Zhang, Tania Tsatralis, Zoe E. Clayton, Laura Z. Vanags, Stacy Robertson, Stephen J. Nicholls, Martin K. C. Ng, Christina A. Bursill, Joanne T. M. Tan

**Affiliations:** 1 The Heart Research Institute, Sydney, Australia; 2 The University of Sydney, Sydney Medical School, Sydney, Australia; 3 Heart Health Theme, South Australian Health & Medical Research Institute, Adelaide, Australia; 4 Adelaide Medical School, Faculty of Health & Medical Sciences, The University of Adelaide, Adelaide, Australia; 5 Department of Cardiology, Royal Prince Alfred Hospital, Sydney, Australia

## Abstract

Diabetic vascular complications are associated with impaired ischaemia-driven angiogenesis. We recently found that reconstituted high-density lipoproteins (rHDL) rescue diabetes-impaired angiogenesis. microRNAs (miRNAs) regulate angiogenesis and are transported within HDL to sites of injury/repair. The role of miRNAs in the rescue of diabetes-impaired angiogenesis by rHDL is unknown. Using a miRNA array, we found that rHDL inhibits hsa-miR-181c-5p expression *in vitro* and using a hsa-miR-181c-5p mimic and antimiR identify a novel anti-angiogenic role for miR-181c-5p. miRNA expression was tracked over time post-hindlimb ischaemic induction in diabetic mice. Early post-ischaemia when angiogenesis is important, rHDL suppressed hindlimb mmu-miR-181c-5p. mmu-miR-181c-5p was not detected in the plasma or within HDL, suggesting rHDL specifically targets mmu-miR-181c-5p at the ischaemic site. Three known angiogenic miRNAs (mmu-miR-223-3p, mmu-miR-27b-3p, mmu-miR-92a-3p) were elevated in the HDL fraction of diabetic rHDL-infused mice early post-ischaemia. This was accompanied by a decrease in plasma levels. Only mmu-miR-223-3p levels were elevated in the hindlimb 3 days post-ischaemia, indicating that rHDL regulates mmu-miR-223-3p in a time-dependent and site-specific manner. The early regulation of miRNAs, particularly miR-181c-5p, may underpin the rescue of diabetes-impaired angiogenesis by rHDL and has implications for the treatment of diabetes-related vascular complications.

## Introduction

Diabetic patients suffer from poor outcomes post myocardial infarction due to impaired coronary collateral formation post-occlusion^1^. Diabetics also experience higher rates of peripheral limb ulceration and amputation arising from peripheral vascular disease^2,3^. Despite advances in the treatment of diabetic vascular complications, many patients remain refractory to current treatment approaches, highlighting the need for alternate therapies. The clinical severity of occlusive arterial disease in diabetic patients has, in part, been attributed to impaired ischaemia-driven angiogenesis, which involves a complex orchestration of signaling pathways and cellular events, beginning with the induction of the hypoxic transcription factor HIF-1α, which promotes the expression of VEGFA, a potent angiogenic mediator. In diabetes, however, HIF-1α stability^4,5^ and VEGFA production and signalling sensitivity^6^ are suppressed, causing the angiogenic response to ischaemia to be impaired.

MicroRNAs (miRNAs) are small non-coding RNAs that post-transcriptionally regulate gene expression by targeting mRNAs causing either partial or complete translational repression. miRNAs can simultaneously control multiple genes; therefore the modulation of a single miRNA has the ability to correct complex diseases^7^. This makes miRNA modulation potentially more powerful than single gene targeting strategies. Circulating miRNAs have emerged as novel biomarkers in angiogenesis-associated diseases, such as cancer and cardiovascular disease (CVD)^8–12^. miRNAs have been implicated in HIF-1α-dependent angiogenic regulation^13^ and can drive pro- or anti-angiogenic effects depending on the downstream targets. miR-27b, for example, is an established pro-angiogenic miRNA in CVD and cancer^14^, while miR-223 has anti-angiogenic properties via regulation of the RPS6KB1/HIF-1α pathway^15^. These studies highlight the potential of miRNAs to act as molecular therapeutic targets for complex diseases such as diabetes-impaired angiogenesis.

HDL has potent anti-diabetic properties and is associated with reduced diabetic vascular complications. Low HDL levels are an independent risk factor for the development of type 2 diabetes mellitus (T2DM)^16^ and are associated with an increased risk of microvascular disease in T2DM patients^17^. We have previously shown that HDL augments ischaemia-driven angiogenesis^18,19^, an effect that is retained in aged mice^20^. We recently discovered that rHDL rescues diabetes-impaired angiogenesis through its ability to increase HIF-1α stability and VEGFA production^21^. The mechanism by which rHDL regulates angiogenesis in diabetes still remains to be fully elucidated, although miRNAs present as highly likely contributors to these effects. Furthermore, HDL is known to transport endogenous miRNAs, delivering them to recipient cells to cause significant functional effects^22,23^.

Accordingly, we sought to investigate the regulation of miRNAs by rHDL in diabetes-impaired angiogenesis. Using a miRNA array, we initially identified 4 miRNAs that were regulated by rHDL. *In vitro* validation studies then revealed a novel anti-angiogenic role for hsa-miR-181c-5p. *In vivo* studies in diabetic mice tracked the miRNA expression over time following the induction of hindlimb ischaemia. It was found that mmu-miR-181c-5p expression in the hindlimb was inhibited early post-ischaemia in diabetic mice infused with rHDL. Whilst mmu-miR-181c-5p was not detected in the HDL fraction of the plasma, mmu-miR-223-3p, mmu-miR-27b-3p and mmu-miR-92a-3p were all elevated in the HDL fraction in rHDL-infused mice early post-ischaemia. mmu-miR-223-3p levels were also elevated in the ischaemic tissue mid-phase post-ischaemia, suggesting rHDL infusions regulate mmu-miR-223-3p in both a time-dependent and site-specific manner. Taken together, our studies show that early regulation of miRNAs, and in particular miR-181c-5p, may underpin the ability of rHDL to rescue diabetes-impaired angiogenesis.

## Results

### Identification of miRNAs involved in the angiogenic action of rHDL

**Figure 1:**
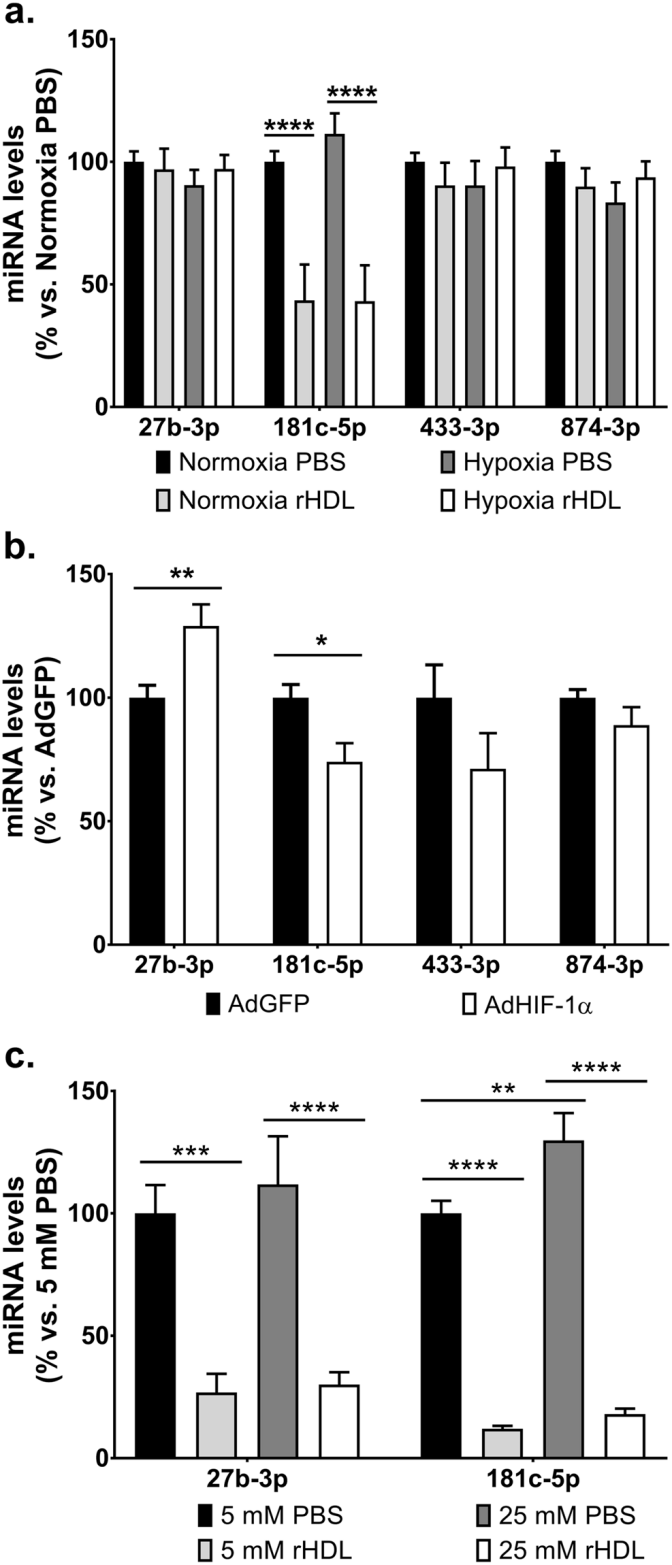
Identification of miRNAs involved in the angiogenic action of rHDL. The expression levels of miRNAs were assessed by qRT-PCR in HCAECs that were either (**a**) treated with rHDL (20 μM, final apoA-I concentration) or PBS (vehicle) prior to normoxia/hypoxia exposure; (**b**) transduced with adenovirus (1 × 10^6^ viral particles/mL) overexpressing HIF-1α (AdHIF-1α) or GFP (AdGFP, adenoviral control) for 48 hours; or (**c**) treated with rHDL (20 μM) or PBS (vehicle) then exposed to glucose conditions (5–25 mM, media supplemented with D-glucose) for 48 hours. Relative changes in miRNA levels were normalised using the ^ΔΔ^*Ct* method to RNU48. Results shown are the mean ± SEM of three independent experiments. *P < 0.05, **P < 0.01, ***P < 0.001, ****P < 0.0001.

We have previously shown that rHDL conditionally regulates angiogenesis, inhibiting inflammatory-driven angiogenesis^18^ and augmenting hypoxia-mediated angiogenesis^18,19^. We now sought to determine the role of miRNAs in the angiogenic action of HDL. A global profile of 874 miRNA targets were assessed using the TaqMan low-density arrays (TLDA) in HCMECs treated with rHDL (20 μM) or PBS (vehicle), prior to hypoxic exposure (1% O_2_) or stimulation with the inflammatory cytokine TNFα (0.6 ng/mL). The array data showed the pro-angiogenic miRNA hsa-miR-27b-3p was suppressed by rHDL in both hypoxia and inflammation (Supplemental Fig. 1). In cells exposed to hypoxia, rHDL also suppressed hsa-miR-433-3p and hsa-miR-874-3p, while in inflammation hsa-miR-181c-5p was suppressed. To confirm the array data, we then assessed the levels of these miRNAs in cells treated with rHDL in hypoxia. In contrast to the original TLDA findings, we found that rHDL only suppressed hsa-miR-181c-5p levels (Fig. 1a) but not hsa-miR-27b-3p, hsa-miR-433-3p or hsa-miR-874-3p in both normoxia and hypoxia. When cells were transduced with an adenovirus to overexpress HIF-1α, hsa-miR-27b-3p levels were elevated (Fig. 1b) supporting its known pro-angiogenic role in hypoxia, and hsa-miR-181c-5p levels were inhibited indicating an anti-angiogenic role. Exposure to high glucose (48 h) elevated hsa-miR-181c-5p, but not hsa-miR-27b-3p levels and pre-incubation with rHDL (20 μM) suppressed this induction (Fig. 1c). Both hsa-miR-27b-3p and hsa-miR-181c-5p levels were inhibited by rHDL irrespective of glucose conditions.

### miR-181c-5p has an inhibitory role in angiogenesis

**Figure 2:**
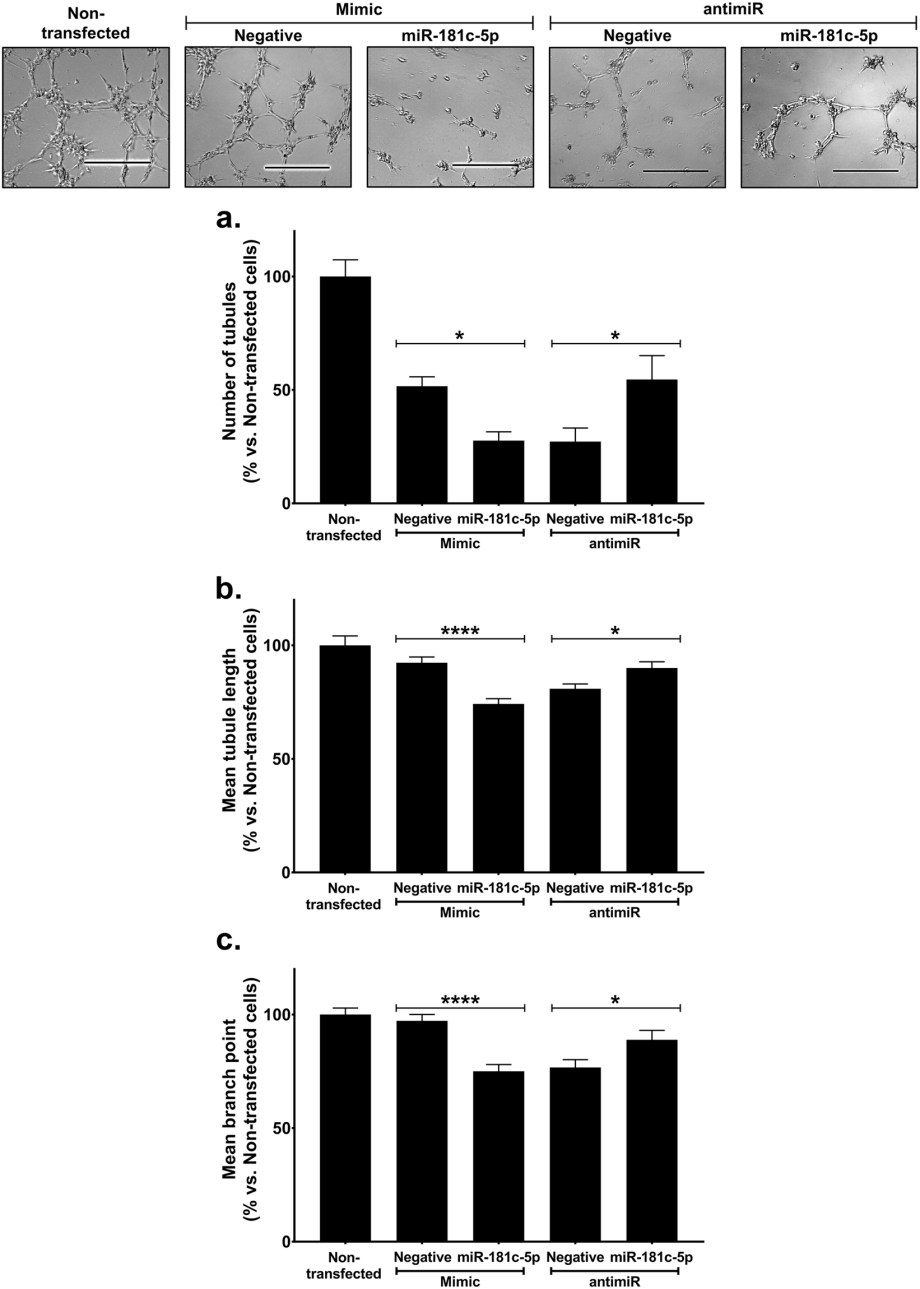
miR-181c-5p has an inhibitory role in angiogenesis. HCAECs were transfected with either hsa-miR-181c-5p mimics (20 nM), antimiRs (100 nM) or appropriate negative controls using Lipofectamine RNAiMax for 6 hours. 48 hours post-transfection, transfected HCAECs were seeded at 8 × 10^3^ cells/well (5 wells/condition) on polymerised growth factor reduced Matrigel and incubated for 5–6 hours. Non-transfected cells were included in the Matrigel assay. The entire well (8–12 fields of view) was photographed at 5X magnification on multiple focal fields under light microscopy and analysed using ImageJ (National Institutes of Health) for (**a**) number of tubules, (**b**) mean tubule length and (**c**) mean branch point. *P < 0.05, ****P < 0.0001.

While miR-27b is an established pro-angiogenic miRNA^14,24^, very little is known about the role of miR-181c-5p in angiogenesis. To directly assess the effects of miR-181c-5p on angiogenesis, cells were transfected with either hsa-miR-181c-5p mimics or antimiRs to overexpress or inhibit hsa-miR-181c-5p (Supplemental Fig. 2). 48 hours post-transfection, cells were used for an *in vitro* tubulogenesis assay. We found that endothelial cells overexpressing hsa-miR-181c-5p had a reduced capacity to form tubules while conversely, when hsa-miR-181c-5p was inhibited, the cells had an increased capacity for tubule formation when compared to their respective transfected negative controls (Fig. 2a). Furthermore, the mean tubule length in cells with increased hsa-miR-181c-5p activity is shorter compared to the negative mimic control (Fig. 2b). However, when hsa-miR-181c-5p activity is inhibited, these cells formed longer tubules compared to the negative antimiR control. Finally, the mean number of branch points were also reduced in hsa-miR-181c-5p mimic cells while hsa-miR-181c-5p antimiR cells had more branch points (Fig. 2c).

**Figure 3:**
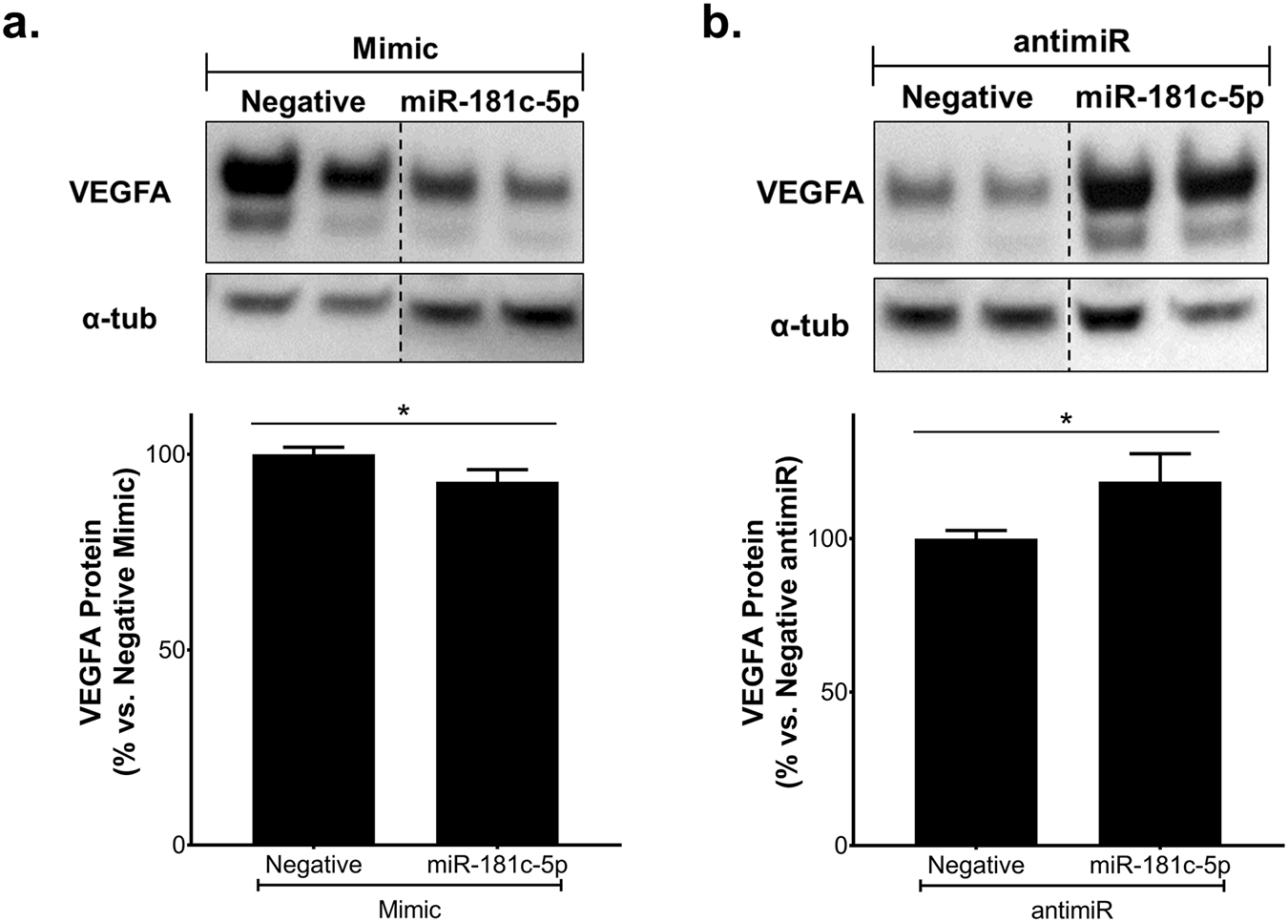
miR-181c-5p regulates VEGFA protein levels. HCAECs were transfected with either hsa-miR-181c-5p mimics (20 nM), antimiRs (100 nM) or appropriate negative controls using Lipofectamine RNAiMax for 6 hours. 48 hours post-transfection, protein lysates were isolated and VEGFA protein levels were measured by Western blot in cells transfected with (**a**) mimics or (**b**) antimiRs. The cropped blots are used in the figure, and the full-length blots are presented in Supplemental Fig. 3. Results shown are the mean ± SEM of three independent experiments. *P < 0.05.

Consistent with these findings, we found that overexpression of hsa-miR-181c-5p caused a small but significant reduction in VEGFA protein levels (Fig. 3a) while inhibition of hsa-miR-181c-5p elevated VEGFA protein (Fig. 3b). Interestingly, *VEGFA* mRNA levels were increased in cells transfected with either hsa-miR-181c-5p mimics or antimiRs (Supplemental Fig 4a and b). To determine if VEGFA is a direct target of hsa-miR-181c-5p, luciferase reporter studies were performed. We found that there was increased luciferase activity in cells co-transfected with the 3′UTR of VEGFA and hsa-miR-181c-5p mimics (Supplemental Fig. 4c); however, no differences were observed when hsa-miR-181c-5p was inhibited (Supplemental Fig. 4d). Sequence alignment of 3′UTR of *VEGFA* and hsa-miR-181c-5p seed sequence reveals that there is a low level of consensus with *VEGFA* (Supplemental Fig. 4e). These findings suggest that the effect of hsa-miR-181c-5p on VEGFA expression is not at a direct transcriptional level but may be indirectly influenced at a translational level.

### rHDL augments diabetes-impaired blood flow perfusion and neovascularisation

**Figure 4:**
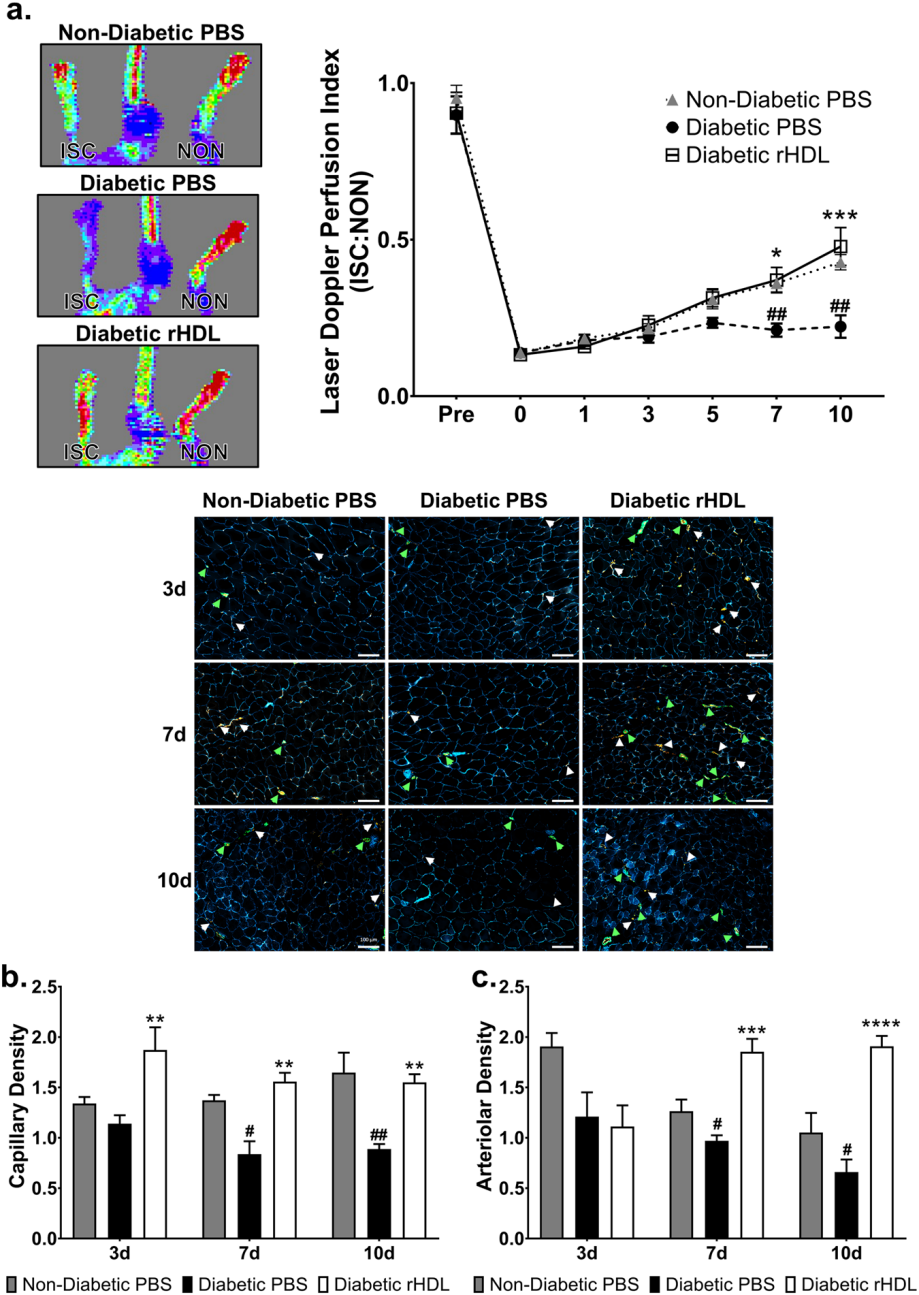
rHDL augments diabetes-impaired blood flow perfusion and neovascularisation. Non-diabetic and diabetic C57Bl/6 J mice underwent femoral artery and vein ligation. Mice received *i.p*. injections of rHDL (400 μg/injection, final apoA-I concentration) or PBS (vehicle) on alternate days one week prior to surgery until sacrifice. (**a**) Hindlimb blood reperfusion was determined by laser Doppler perfusion imaging; representative images show high (red) to low (blue) blood flow at Day 10. Laser Doppler perfusion index was determined as a ratio of the ischaemic (ISC): non-ischaemic (NON) hindlimb. Grey triangles, Non-Diabetic PBS mice; black circles, Diabetic PBS mice; white squares, Diabetic rHDL mice. Gastrocnemius muscle sections were stained for capillaries (CD31^+^, stained red, denoted by white arrows), arterioles (SMC α-actin^+^, stained green, denoted by green arrows) and myocytes (laminin^+^, stained blue). Scale bar: 100 μm. (**b**) Capillary density (CD31^+^/myocytes) and (**c**) arteriolar density (SMC α-actin^+^/myocytes) was determined as a ratio of ischaemic: non-ischaemic hindlimb. Results shown are the mean ± SEM (n = 5–6/group). ^#^*P* < 0.05, ^##^*P* < 0.01 vs. Non-Diabetic PBS; *P < 0.05, **P < 0.01, ***P < 0.001, ****P < 0.0001 vs. Diabetic PBS.

We next assessed the regulation of miRNAs by rHDL *in vivo* using the hindlimb ischaemia model of ischaemia-mediated angiogenesis. Consistent with our previous findings^21^, rHDL infusions rescued diabetes-impaired blood flow perfusion, a marker of angiogenesis, in streptozotocin-rendered diabetic mice (Fig. 4a). The effects of rHDL were independent of changes in glucose and lipid levels (Supplemental Tables 1 and 2). 3 days post-ischaemic induction, diabetic rHDL-infused mice had higher capillary density compared to the diabetic PBS mice (Fig. 4b), however there was no difference between the non-diabetic and diabetic PBS animals. Diabetes impaired capillary formation at the later stages (days 7 and 10) post-ischaemic induction. However, an increase in the number of capillaries formed per myocyte was still observed in diabetic animals treated with rHDL. The number of arterioles formed were also elevated in these animals in the mid-late stages post-ischaemia (Fig. 4c).

### mmu-miR-181c-5p is suppressed by rHDL early post-ischaemia in diabetic mice

Studies have reported the temporal expression of miRNAs in angiogenesis^25^. miRNA levels were measured at the site of ischaemia, within the HDL fraction and in the plasma in cohorts of mice at time points reflective of early (0–24 h), mid (3–7d) and late (10d) stages of angiogenesis to determine if the pro-angiogenic effects of HDL in diabetes *in vivo* is facilitated by regulation of miRNAs at the site of ischaemic injury or within the HDL particle.

**Figure 5:**
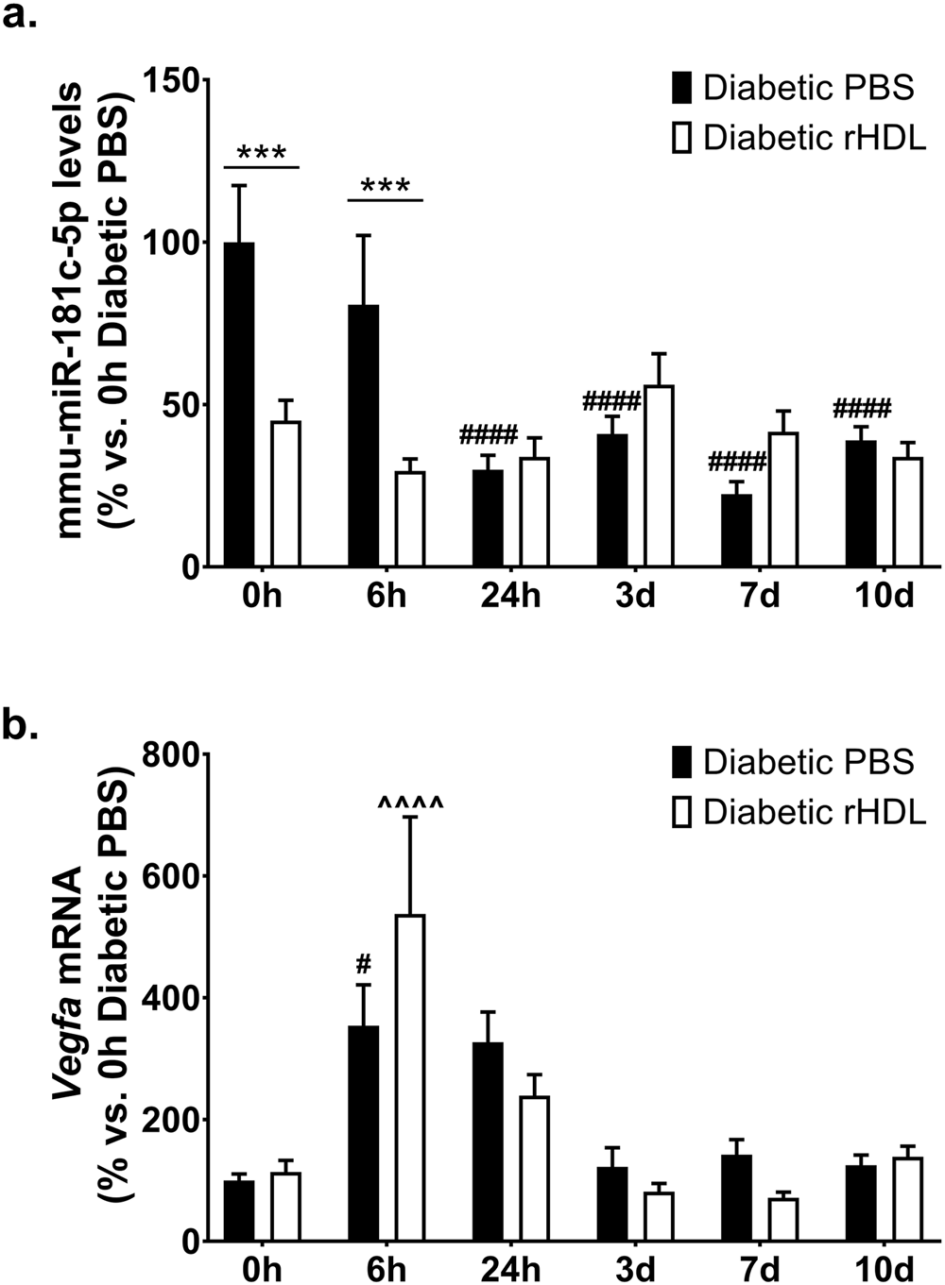
mmu-miR-181c-5p is suppressed by rHDL early post-ischaemia in diabetic mice. Diabetic C57Bl/6 J mice received *i.p*. injections of rHDL (400 μg/injection, final apoA-I concentration) or PBS (vehicle) on alternate days one week prior to surgery until sacrifice. (**a**) Hindlimb mmu-miR-181c-5p levels, normalised to RNU68. (**b**) Hindlimb *Vegfa* mRNA levels, normalised to *36B4*. ***P < 0.001 vs. relative timepoint Diabetic PBS; ^#^*P* < 0.05 ^####^*P* < 0.001 vs. 0 h Diabetic PBS; ^^^^*P* < 0.0001 vs. 0 h Diabetic rHDL.

We firstly tracked the expression levels of our novel anti-angiogenic miRNA, mmu-miR-181c-5p in the ischaemic hindlimb. Early post-ischaemia (6 h) when the induction of angiogenesis is most important, rHDL suppressed hindlimb mmu-miR-181c-5p levels (Fig. 5a), which were paralleled with an elevation in *Vegfa* levels (Fig. 5b). mmu-miR-181c-5p was not detected in the HDL fraction or in the plasma, suggesting that the effects of rHDL on mmu-miR-181c-5p is focussed at the site of ischaemia.

### rHDL increases mmu-miR-223-3p levels in the HDL particle 6 hours post-ischaemia and augments hindlimb mmu-miR-223-3p levels 3 days post-ischaemia

**Figure 6:**
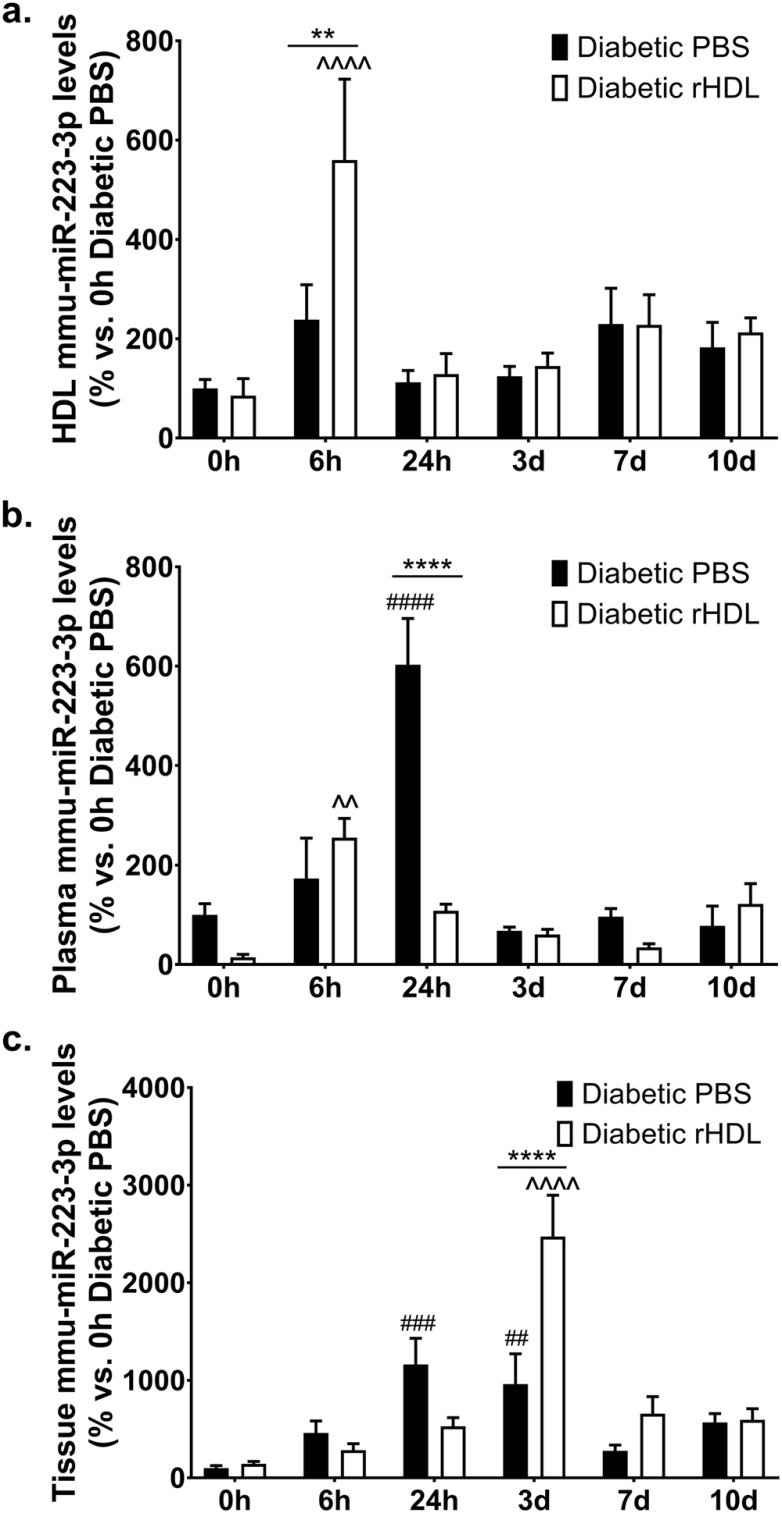
rHDL increases mmu-miR-223-3p levels in the HDL particle 6 hours post-ischaemia and augments hindlimb mmu-miR-223-3p levels 3 days post-ischaemia. Diabetic C57Bl/6 J mice received *i.p*. injections of rHDL (400 μg/injection, final apoA-I concentration) or PBS (vehicle) on alternate days one week prior to surgery until sacrifice. mmu-miR-223-3p levels, normalised to RNU68 in the (**a**) HDL fraction, (**b**) plasma and (**c**) tissue. Results shown are the mean ± SEM (n = 5–6/group). **P < 0.01, ****P < 0.0001 vs. relative timepoint Diabetic PBS; ^##^*P* < 0.01, ^###^*P* < 0.001, ^####^*P* < 0.0001 vs. 0 h Diabetic PBS; ^^*P* < 0.01, ^^^^*P* < 0.0001 vs. 0 h Diabetic rHDL.

In addition to mmu-miR-181c-5p, we also determined the effect of rHDL on three other miRNAs: mmu-miR-223-3p, mmu-miR-27b-3p and mmu-miR-92a-3p. These miRNAs were selected based on previous evidence for either a role in angiogenesis or their presence and transportation in HDL. HDL transport of endogenous miRNAs is a known mechanism for miRNA delivery to recipient cells with functional targeting capabilities^23^. We first assessed the expression levels of mmu-miR-223-3p, an anti-angiogenic miRNA^26^ that has previously been shown to be carried by HDL^23^. Six hours post-ischaemic induction, there was a 5.8-fold increase in the levels of mmu-miR-223-3p within the HDL fraction of diabetic mice infused with rHDL (Fig. 6a). Plasma mmu-miR-223-3p levels in the diabetic PBS infused mice were significantly elevated 24 hours post-ischaemia. This elevation in plasma mmu-miR-223-3p levels did not occur in mice infused with rHDL (Fig. 6b). Whilst diabetic PBS control animals had elevated hindlimb mmu-miR-223-3p levels in the early-mid stages (24 h and day 3) post-ischaemia, compared to the 0 h timepoint (Fig. 6c), infusions of rHDL caused a substantial 2.5-fold increase in mmu-miR-223-3p levels in the tissue at day 3. This may suggest that infusions of rHDL are increasing hindlimb levels of the anti-angiogenic miRNA mmu-miR-223-3p at a timepoint in which the initial need for an induction in angiogenesis was starting to decline and the remodelling events were commencing.

### rHDL induces mmu-miR-27b-3p and mmu-miR-92a-3p levels in the HDL particle 6 hours post-ischaemia

**Figure 7:**
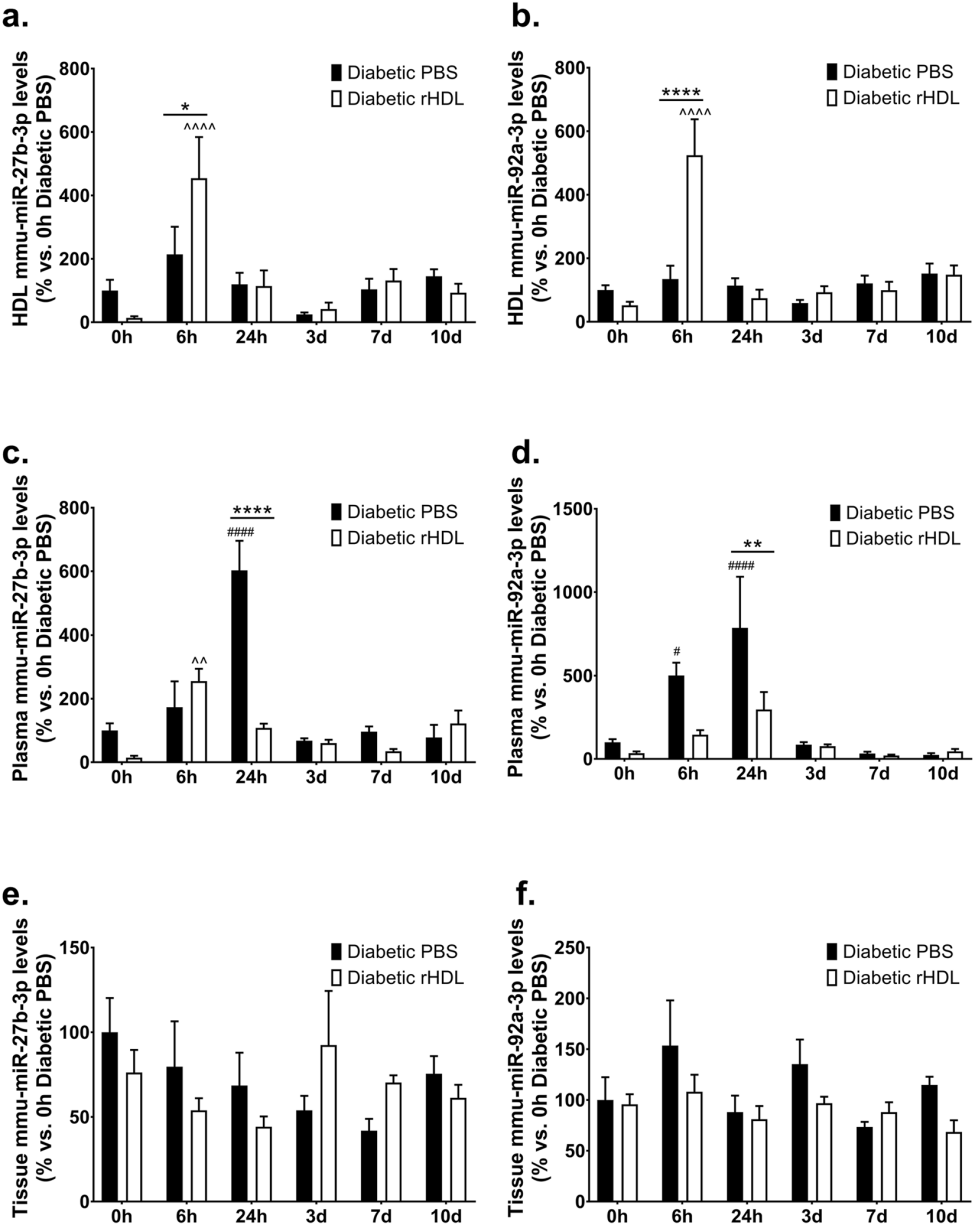
rHDL induces mmu-miR-27b-3p and mmu-miR-92a-3p levels in the HDL particle 6 hours post-ischaemia. Diabetic C57Bl/6 J mice received *i.p*. injections of rHDL (400 μg/injection, final apoA-I concentration) or PBS (vehicle) on alternate days one week prior to surgery until sacrifice. mmu-miR-27b-3p and mmu-miR-92a-3p levels, normalised to RNU68 in the (**a,b**) HDL fraction, (**c,d**) plasma and (**e,f**) tissue respectively. Results shown are the mean ± SEM (n = 5–6/group). *P < 0.05, **P < 0.01, ****P < 0.0001 vs. relative timepoint Diabetic PBS; ^#^*P* < 0.05, ^####^*P* < 0.0001 vs. 0 h Diabetic PBS; ^^*P* < 0.01, ^^^^*P* < 0.0001 vs. 0 h Diabetic rHDL.

We then sought to determine if rHDL also regulates two other angiogenic miRNAs: mmu-miR-27b-3p, a pro-angiogenic miRNA^24^ that was identified in the original miRNA profile study to be regulated by rHDL and mmu-miR-92a-3p, an established anti-angiogenic miRNA^27^. rHDL promoted an increase in both mmu-miR-27b-3p (Fig. 7a) and mmu-miR-92a-3p (Fig. 7b) levels within the HDL fraction 6 hours post-ischaemia. A decrease in plasma miRNA levels was observed in rHDL-treated mice at the 24-hour timepoint (mmu-miR-27b-3p: Fig. 7c and mmu-miR-92a-3p: Fig. 7d). However, no differences were observed in tissue miRNA levels for both these miRNAs (Fig. 7e and f). Our findings suggest that whilst rHDL is able to increase the levels of mmu-miR-27b-3p and mmu-miR-92a-3p in the HDL fraction, the lack of change at the ischaemic site implies they do not play a significant role in the augmentation of angiogenesis in diabetes by rHDL.

## Discussion

We have previously reported that rHDL rescues diabetes-impaired angiogenesis via salutary effects on the classical HIF-1α/VEGFA angiogenic signalling pathway^21^, however, the involvement of miRNAs is unknown. This study identified and confirmed a novel anti-angiogenic role for miR-181c-5p using mimics and antimiRs, which is also inhibited by rHDL *in vitro*. Infusions of rHDL to diabetic mice suppressed mmu-miR-181c-5p levels in the hindlimb tissue in the first 6 hours post-ischaemia. Analysis of other angiogenic miRNAs revealed that rHDL infusions increased mmu-miR-223-3p levels in the HDL fraction 6 hours post-ischaemia and in the hindlimb 3 days post-ischaemia. This suggests that rHDL infusions influence differential and site-specific expression of mmu-miR-223-3p in a time-dependent manner. The increase in hindlimb mmu-miR-223-3p may serve to suppress the initial induction of angiogenesis once capillary and arteriolar density are sufficient for adequate perfusion. Taken together, these findings support a role for miRNAs, particularly miR-181c-5p, in the rescue of diabetes-impaired angiogenesis by rHDL. Inhibition of miR-181c-5p may present as a potential therapeutic strategy for the treatment of diabetes-related vascular complications.

We have previously found that rHDL augments hypoxia-induced angiogenesis^18,19^. Using a miRNA array, we identified 4 miRNAs (hsa-miR-27b-3p, hsa-miR-181c-5p, hsa-miR-433-3p and hsa-miR-874-3p), that were regulated by rHDL. However, only hsa-miR-27b-3p and hsa-miR-181c-5p were regulated by HIF-1α overexpression and suppressed by rHDL in high glucose. While miR-27b-3p is an established pro-angiogenic miRNA^14,24^, miR-181c-5p had no known role in angiogenesis. To date, studies on miR-181c have focused on its link to Alzheimer’s disease and cancer progression^28^. miR-181c-modulated genes converge on signalling cascades relevant to neurite and synapse development, suggesting a role in cortical neuronal maturation^29^. Consistent with a role in angiogenesis, miR-181c is implicated in the development and progression of pancreatic cancer^30^ and inflammatory breast cancer^31^. Furthermore, differential profiling of miRNAs in placental development found that miR-181c was significantly upregulated in third trimester placentas^32^. This study has directly demonstrated for the first time that miR-181c-5p is an anti-angiogenic miRNA, where overexpression of hsa-miR-181c-5p inhibited endothelial vascular network formation while hsa-miR-181c-5p inhibition promoted tubule formation *in vitro*. *In vivo*, rHDL suppressed mmu-miR-181c-5p locally in the ischaemic hindlimb tissue of diabetic mice in the early phase post-ischaemia. mmu-miR-181c-5p levels were also reduced 24 hours post-ischaemia in the diabetic PBS animals, suggesting that miR-181c-5p suppression is critical to augment angiogenesis in the early stages post-ischaemia when the need for angiogenesis is greatest. The earlier suppression of mmu-miR-181c-5p by rHDL in the ischaemic tissue may trigger a more rapid angiogenic response to the ischaemic stimulus. mmu-miR-181c-5p expression was not detected in either the HDL fraction or in the circulation, suggesting that the regulation of mmu-miR-181c-5p expression by rHDL is restricted to the site of tissue ischaemia.

This study also explored the effect of rHDL on three established angiogenic miRNAs: miR-223-3p, miR-27b-3p and miR-92a-3p. HDL transport of endogenous miRNAs is a mechanism for their delivery to recipient cells with functional targeting capabilities^23^. Infusions of rHDL into mice causes distinctly different miRNA profiles between normal and atherogenic models^23^. miR-223 was found to be the most abundant HDL-associated miRNA in familial hypercholesterolaemia^23^ and its delivery to endothelial cells was shown to confer anti-inflammatory effects^22^. In the current study, mmu-miR-223-3p levels were elevated in the HDL fraction of diabetic rHDL-infused mice 6 hours post-ischaemia, which was accompanied with a reduction in circulating plasma levels at 24 hours. Animals treated with rHDL had elevated hindlimb mmu-miR-223-3p levels at the mid-phase post-ischaemia (3 days). Previous studies have shown that miR-223 is an anti-angiogenic miRNA, where overexpression of miR-223 suppressed angiogenic pathways including VEGFA stimulated phosphorylation of Akt, proliferation and migration^15,26^. The anti-angiogenic effects of miR-223 were also demonstrated *in vivo* with enhanced perfusion recovery seen in miR-223^−/y^ mice post-ischaemic induction^26^. We postulate that the increase in hindlimb mmu-miR-223-3p levels seen in the HDL-treated mice may serve as a negative regulator of the angiogenic response during the mid-stage post-ischaemia, when the early-stage induction of neovascularisation is complete and a tapering of pro-angiogenic processes is required as the tissue remodelling phase approaches.

Our miRNA profile array found that rHDL suppressed hsa-miR-27b-3p in high glucose conditions *in vitro*. miR-27b was shown to improve tissue revascularisation and blood flow perfusion in a non-diabetic mouse model of critical limb ischaemia^14^ and augmented wound perfusion, capillary formation and healing in diabetic mice^33^. As rHDL rescues both diabetes-impaired ischaemia-induced neovascularisation and wound angiogenesis and healing^21^, we proposed that miR-27b may mediate the pro-angiogenic effects of rHDL. miR-92a is an established anti-angiogenic miRNA^34^, with pre-clinical studies showing that miR-92a inhibition enhanced blood vessel growth and functional recovery of damaged tissue in mouse models of limb ischaemia and myocardial infarction^27^. While rHDL increased both mmu-miR-27b-3p & mmu-miR-92a-3p levels in the HDL particle, there was no change at the ischaemic site, suggesting that these miRNAs may not be critical in the action of rHDL in a diabetic milieu. The importance of elevating mmu-miR-27b-3p and mmu-miR-92a-3p in the HDL fraction with no change at the site of ischaemia is unclear. It may suggest that these miRNAs are delivered to other sites such as the liver, the organ that HDL is metabolised through, with studies demonstrating hepatic miR-27b expression is responsive to lipid levels and controls multiple genes critical to dyslipidaemia^23^.

This is the first study to comprehensively track over time the regulation of miRNAs in the rescue of diabetes-impaired angiogenesis by rHDL. We found that the regulation of the miRNAs by rHDL occurs early post-ischaemia at the tissue site and in the HDL fraction. We posit that this early regulation by rHDL, particularly the suppression of mmu-miR-181c-5p at the ischaemic tissue site may, at least in part, contribute to the pro-angiogenic actions of HDL in diabetes. A key hallmark of diabetes impaired neovascularisation is reduced VEGFA production and sensitivity^6^. We previously found that rHDL rescues diabetes-impaired neovascularisation by augmenting VEGFA production and VEGFR2 signalling^21^. It has been previously reported that overexpression of two VEGFA isoforms (VEGFA165 and VEGFA206) resulted in the downregulation of miR-181c expression in a lung adenocarcinoma line *in vitro*^35^ but whether VEGFA is a downstream target of miR-181c is unknown. Cells transfected with either hsa-miR-181c-5p mimics or antimiRs had small but significant differences in VEGFA protein levels. However, qPCR and luciferase assays coupled with sequence alignment showed that VEGFA is not a direct transcriptional target of hsa-miR-181c-5p. Bioinformatics miRNA pathway analysis using DIANA-mirPath^36^ encompassing 3 databases (TargetScan, microT-CDS and Tarbase) predicted that miR-181c-5p targets genes that contribute to key cellular processes involved in angiogenesis including NOS regulation, TGF-β1 signalling, FGF signalling and post-translational protein modification. Taken together, we postulate that the effect of miR-181c-5p on VEGFA expression is not at a direct transcriptional level but may be indirectly influenced at a translational level by alternate signalling pathways. Furthermore, VEGFA mediates its pro-angiogenic effects by binding to VEGFR2 to trigger autophosphorylation of the receptor, activating downstream intracellular pathways which ultimately contribute to angiogenesis^37^. Therefore, we believe that the small translational effects of mir-181c-5p on VEGFA protein could have striking downstream effects on angiogenesis. Previous studies have found that miR-181c acts as a control point in the phosphorylation of Akt^38^, a key signalling protein for angiogenesis. rHDL augments the Akt signalling pathway in hypoxia^39^ and high glucose^21^. Furthermore, inhibition of the Akt signalling pathway abrogated the pro-angiogenic effects of rHDL in hypoxia^19^. These studies add further support for a role of miR-181c-5p in angiogenesis and suggest that the downstream targets of miR-181c-5p may not be restricted to just VEGFA but other critical angiogenic signalling proteins.

**Figure 8:**
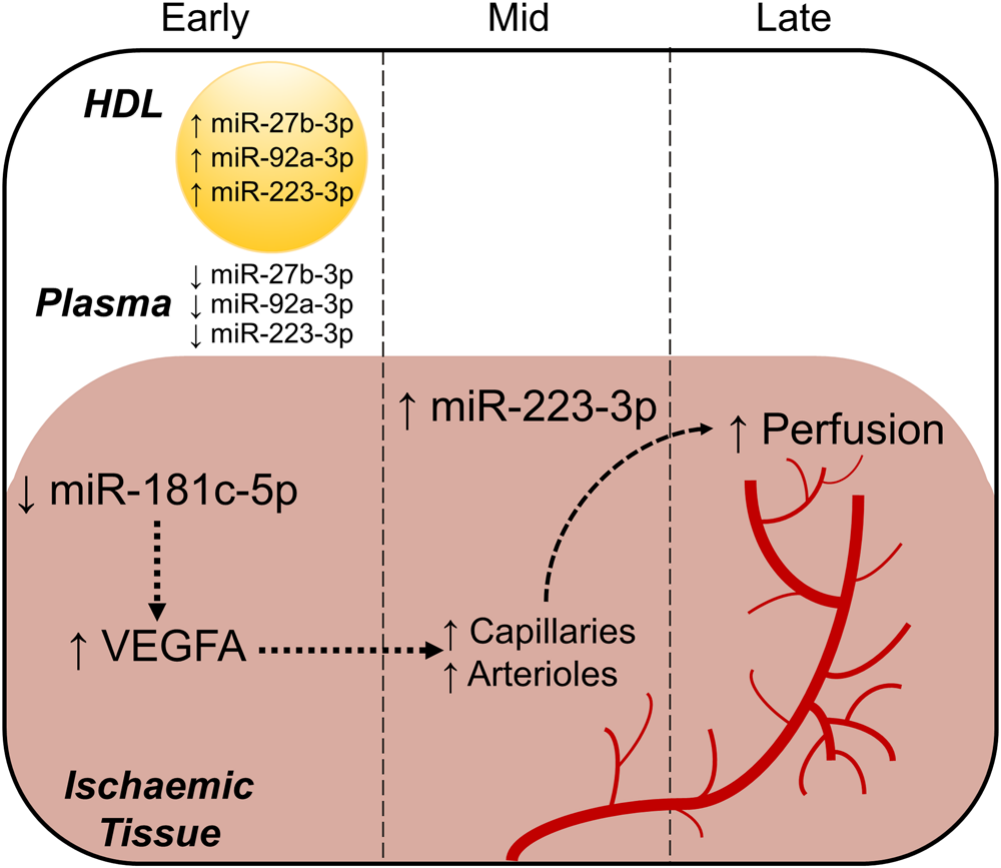
The regulation of miRNAs by rHDL in diabetes-impaired angiogenesis. rHDL induces changes in the expression of miRNAs in diabetic mice. These changes predominantly occur in the early phase post-ischaemic induction where (1) mmu-miR-181c-5p levels are suppressed in the ischaemic hindlimb; (2) mmu-miR-223-3p, mmu-miR-27b-3p and mmu-miR-92a-3p are elevated in the HDL fraction; and (3) plasma mmu-miR-223-3p, mmu-miR-27b-3p and mmu-miR-92a-3p levels are reduced. However, only mmu-miR-223-3p levels were elevated at the ischaemic site in the mid-stage post-ischaemia, which may serve to suppress the initial induction of angiogenesis once capillary and arteriolar density have reached levels sufficient to restore adequate perfusion. *In vitro* gain-of-function (mimics) and loss-of-function (antimiRs) studies suggest that miR-181c-5p may have indirect effects on VEGFA at a translational level, supporting an anti-angiogenic role for miR-181c-5p in angiogenesis, which may contribute towards increased neovascularisation in the mid phase post-ischaemia and culminating in blood flow perfusion at the later stage. ↑ and ↓ denotes the effects of rHDL.

In conclusion, we have identified a novel anti-angiogenic miRNA, miR-181c-5p, first using array analysis and confirmed using hsa-miR-181c-5p mimics and antimiRs. We have tracked changes in miRNA expression in diabetic mice in response to ischaemia and tested the effect of rHDL infusions (Fig. 8). We find changes in miRNAs and they predominantly occur in the early phase post-ischaemia where infusions of rHDL cause: (1) a suppression in mmu-miR-181c-5p levels in the ischaemic hindlimb, consistent with an anti-angiogenic role for miR-181c-5p; (2) an elevation in mmu-miR-223-3p, mmu-miR-27b-3p and mmu-miR-92a-3p levels in the HDL fraction; and a (3) reduction in mmu-miR-223-3p, mmu-miR-27b-3p and mmu-miR-92a-3p plasma levels. However, only mmu-miR-223-3p levels were elevated at the ischaemic site in the mid-stage post-ischaemia. Elevated mmu-miR-223-3p during the mid-ischaemic phase may serve to suppress the initial induction of angiogenesis once capillary and arteriolar density have reached levels sufficient to restore adequate perfusion. This study provides a greater understanding of the mechanisms by which rHDL rescues diabetes-impaired angiogenesis; particularly the involvement of miRNAs, which may provide alternate therapeutic targets for the improvement of diabetes-associated vascular complications and the translation of HDL.

## Methods

### Preparation of discoidal reconstituted HDL

apoA-I was isolated from pooled samples of multiple donated plasma samples from healthy humans obtained from the Australian Red Cross (Supply Agreement 14-02NSW-04) by ultracentrifugation and anion-exchange chromatography as described previously^18,21^. Approval was granted by the Sydney Local Health District Human Ethics Review Committee (HREC\EXECOR\15-06) and conformed to the Declaration of Helsinki, with informed consent obtained at the time of collection. Discoidal reconstituted HDL (rHDL) was prepared by complexing apoA-I with 1-palmitoyl-2-linoleoyl-phosphatidylcholine (PLPC).

### Profiling of miRNAs

Human cardiac microvascular endothelial cells (HCMECs, PromoCell) were cultured in EGM-2MV media and used at passage 5. Cells were plated at 8 × 10^4^ cells/well and grown to 60% confluency. HCMECs were treated with rHDL (20 μM, final apoA-I concentration) or PBS (vehicle control) for 24 hours then exposed to two forms of angiogenic stimuli: (1) hypoxia (1% O_2_ balanced with N_2_) for 8 hours; or (2) stimulation with the inflammatory cytokine TNFα (0.6 ng/mL) for 4.5 hours. Two donors were used and the experiments were performed independently 5 times.

Expression of 874 miRNAs was performed by reverse transcription and quantitative real-time PCR (qPCR) using the Megaplex TaqMan Low Density Human miRNA Array (TLDA). Total RNA was extracted using TRI reagent. 1000 ng total RNA was retro-transcribed using stem-loop primers and the amplified product was loaded onto the TLDA and signals were detected by qPCR. The fold change of miRNAs was calculated using the comparative Cq method (2^−ΔΔCq^) and normalised to endogenous U6 internal reference gene.

### Validation of miRNA profiling data

Human coronary artery endothelial cells (HCAECs, Cell Applications) were cultured in MesoEndo growth medium and used at passages 4–6. To confirm the miRNA profiling data, cells were either (1) treated with rHDL (20 μM, final apoA-I concentration) or PBS (vehicle) prior to normoxia/hypoxia exposure; (2) transduced with adenovirus (1 × 10^6^ viral particles/mL) overexpressing HIF-1α (AdHIF-1α) or GFP (AdGFP, adenoviral control) for 48 hours; or (3) treated with rHDL (20 μM) or PBS (vehicle) then exposed to glucose conditions (5–25 mM, media supplemented with D-glucose) for 48 hours. Each experiment was conducted three times independently in triplicate.

### MicroRNA-181c-5p transfection

HCAECs were seeded at 1.5 × 10^5^ cells/well and grown to 80% confluency. HCAECs were transfected with hsa-miR-181c-5p mimics (20 nM), antimiRs (100 nM) or appropriate controls using Lipofectamine RNAiMax for 6 hours. 48 hours post-transfection, cells were assessed for Matrigel tubulogenesis assays, VEGFA protein and *VEGFA* mRNA levels. Each experiment was conducted five times independently in triplicate.

### Matrigel tubulogenesis assay

Transfected HCAECs were seeded at 8 × 10^3^ cells/well (5 wells/condition) on polymerised growth factor reduced Matrigel and incubated for 5–6 hours. Non-transfected cells were included in the Matrigel assay. The entire well (8–12 fields of view) was photographed at 5X magnification on multiple focal fields under light microscopy and analysed using ImageJ (National Institutes of Health) for number of tubules, mean tubule length and mean branch point.

### Western blot

Protein was extracted using RIPA lysis buffer^21^ and subjected to Western blot analysis and probed with VEGFA antibody (ab46154, Abcam). Even protein loading was confirmed by α-tubulin (ab40742, Abcam).

### Luciferase reporter assay

Ad293 cells were co-transfected with 150 ng luciferase reporter construct containing 3′-untranslated region (3′UTR) of VEGFA (GeneCopoeia) and with either hsa-miR-181c-5p mimics, antimiRs or respective negative controls. Luciferase activity was measured 48 hours after transfection using the Secrete-Pair™ Dual Luminescence Assay Kit.

### Animals

All experimental procedures were conducted with approval from the Sydney Local Health District Animal Welfare Committee (#2015/038) and conformed to the Guide for the Care and Use of Laboratory Animals (United States National Institute of Health). 8-week-old male C57BL/6J mice were rendered diabetic 2 weeks prior to surgery by a bolus *i.p*. injection of streptozotocin (165 μg/g).

### Hindlimb ischaemia model

The hindlimb ischaemia model was conducted as described previously to ensure ischaemia in the distal regions of the hindlimb^18,21,40^. The left femoral artery and vein were ligated and excised proximal to the *profunda femoris* and *epigastrica* arteries and distally to the popliteal fossa^40^. Mice (n = 5–6/group) received three *i.p*. injections of either rHDL (400 μg/injection, final apoA-I concentration) or PBS (vehicle) on alternate days one week prior to surgery until sacrifice. Hindlimb blood reperfusion was determined by laser Doppler perfusion imaging prior to and immediately following surgery and then at days 1, 3, 5, 7, and 10 post-ischaemic induction. Parallel cohorts of mice were assessed at baseline (0 hours), early (6 & 24 hours), mid (3 & 7 days) and later (10 days) time points post-ischaemia.

### Plasma glucose and lipid concentrations

Glucose concentrations were measured using a glucometer (Accu-Chek Performa). Total, HDL, and LDL cholesterol concentrations on mouse plasma were determined enzymatically (Roche Diagnostics). HDL cholesterol concentrations were determined following polyethylene glycol precipitation of apoB-containing lipoproteins^18,21^.

### Immunocytochemistry

Fresh frozen 5-μm sections of gastrocnemius muscle from ischaemic and non-ischaemic hindlimbs of mice at 3, 7 and 10 days post-ischaemia were stained to detect the number of CD31^+^ capillaries (ab25644, Abcam) and α-SMC^+^ arterioles (F3777-2M, Sigma) per myocyte (laminin; Abcam). Three separate regions within each section were imaged at 10X magnification and repeated across 2 slides with 3 sections per sample. All images were then analysed digitally on Image-Pro Premier software to determine capillary density (CD31^+^ capillaries/myocyte) and arteriolar density (α-SMC^+^ arterioles/myocyte).

### RNA extraction, reverse transcription and qRT-PCR

Total RNA was isolated using TRI reagent and up to 1000 ng total RNA was reverse transcribed using the miScript II RT synthesis kit. qPCR was used to determine the expression levels of (1) hsa-miR-27b-3p, hsa-miR-181c-5p, hsa-miR-433-3p and hsa-miR-874-3p in the treated cells, and (2) mmu-miR-181c-5p, mmu-miR-223-3p, mmu-miR-27b-3p and mmu-miR-92a-3p in the HDL fraction, circulating plasma and gastrocnemius muscles using miScript primer assays (Qiagen). Relative changes in miRNA levels were normalised using the ^ΔΔ^*Ct* method to RNU48 for treated cells or RNU68 for the murine samples. qPCR was also performed for (1) *VEGFA* levels (normalised to *B2M*) in the hsa-mir-181c-5p transfected cells and (2) *Vegfa* levels in the gastrocnemius muscles and normalised to murine *36B4* using primers designed previously^21^.

### Statistical analysis

Data are expressed as mean ± SEM. Differences between treatment groups were calculated using a one-way ANOVA (Tukey’s *post hoc* comparison test) or Student’s *t*-test. A two-way ANOVA (Tukey’s *post hoc* comparison test) was performed when comparing data at multiple time points. Significance was set at a two-sided *P* < 0.05.

## Electronic supplementary material

Supplemental Material

## Data Availability

The datasets generated or analysed during the current study are available from the corresponding author on reasonable request.
