# Supplementary material for: The regulation of miRNAs by reconstituted high-density lipoproteins in diabetes-impaired angiogenesis
Source: Sci Rep. 2018 Sep 11;8:13596. doi: 10.1038/s41598-018-32016-x (PMC6133943; doi:10.1038/s41598-018-32016-x)
Supplement: Supplementary file 1 — Supplemental Material [file 41598_2018_32016_MOESM1_ESM.pdf]

Supplementary Information for:

The regulation of miRNAs by reconstituted high-density lipoproteins in diabetes-impaired angiogenesis

Samuel T. Hourigan[1,2], Emma L. Solly[3], Victoria A. Nankivell[3], Anisyah Ridiandries[1,2], Benjamin M. Weimann[3,4], Rodney Henriquez[1], Edward R. Tepper[1,2], Jennifer Q.J. Zhang[1,2], Tania Tsatralis[1], Zoë E. Clayton[1,2], Laura Z. Vanags[1,2], Stacy Robertson[1,2], Stephen J. Nicholls[3,4], Martin K.C. Ng[1,2,5], Christina A. Bursill[1,2,3,4§*], Joanne T.M. Tan[1,2,3,4§*].

[§] Equal author contributions to publication.

[1]The Heart Research Institute, Sydney, Australia; [2]The University of Sydney, Sydney Medical School, Sydney, Australia; [3]Heart Health Theme, South Australian Health & Medical Research Institute, Adelaide, Australia; [4]Adelaide Medical School, Faculty of Health & Medical Sciences, The University of Adelaide, Adelaide, Australia; [5]Department of Cardiology, Royal Prince Alfred Hospital, Sydney, Australia.

Supplemental Table 1

Blood Glucose (mM)	Non-Diabetic PBS	Diabetic PBS	Diabetic rHDL
0 hours	10.58±0.36	33.17±0.13****	31.03±2.27****
6 hours	11.05±0.65	29.02±2.13****	26.45±2.27****
24 hours	10.72±0.81	32.63±0.52****	27.08±4.26****
3 days	9.77±0.41	30.05±1.92****	29.03±2.08****
7 days	14.50±3.79	27.95±3.78****	31.32±1.33****
10 days	10.40±0.65	29.87±2.39****	33.30±0.00****

Supplemental Table 1: Plasma glucose levels.
Data expressed as mean±SEM (n = 6/group). ****P<0.0001 vs. Non-Diabetic PBS by One-way ANOVA, Tukey's comparison test *post hoc*.

Supplemental Table 2

Total Cholesterol (mg/mL)	Non-Diabetic PBS	Diabetic PBS	Diabetic rHDL
0 hours	1.28±0.19	1.37±0.13	1.32±0.16
6 hours	1.47±0.34	1.34±0.13	1.51±0.24
24 hours	1.49±0.17	1.83±0.29	1.53±0.25
3 days	0.91±0.11	1.22±0.15	1.09±0.14
7 days	1.03±0.12	1.16±0.16	1.11±0.12
10 days	0.95±0.09	1.27±0.34	1.08±0.21

HDL Cholesterol (mg/mL)	Non-Diabetic PBS	Diabetic PBS	Diabetic rHDL
0 hours	0.44±0.09	0.41±0.06	0.37±0.04
6 hours	0.44±0.08	0.43±0.05	0.46±0.03
24 hours	0.39±0.04	0.51±0.07	0.43±0.06
3 days	0.32±0.03	0.34±0.04	0.30±0.02
7 days	0.29±0.03	0.35±0.03	0.32±0.02
10 days	0.32±0.02	0.40±0.08	0.32±0.03

LDL Cholesterol (mg/mL)	Non-Diabetic PBS	Diabetic PBS	Diabetic rHDL
0 hours	0.84±0.10	0.96±0.11	0.95±0.13
6 hours	1.03±0.27	0.91±0.13	1.05±0.21
24 hours	1.10±0.15	1.32±0.25	1.10±0.20
3 days	0.59±0.11	0.88±0.13	0.79±0.12
7 days	0.74±0.10	0.80±0.14	0.79±0.11
10 days	0.63±0.07	0.87±0.27	0.74±0.16

Triglycerides (mg/dL)	Non-Diabetic PBS	Diabetic PBS	Diabetic rHDL
0 hours	1.46±0.22	1.54±0.27	1.58±0.25
6 hours	1.14±0.07	1.92±0.25	1.67±0.33
24 hours	1.18±0.08	1.50±0.31	1.43±0.20
3 days	1.43±0.17	1.24±0.13	1.33±0.21
7 days	1.46±0.20	1.88±0.18	1.34±0.23
10 days	1.65±0.16	1.99±0.30	1.15±0.22

Supplemental Table 2: Plasma total cholesterol, HDL and LDL cholesterol levels and triglycerides. Data expressed as mean±SEM (n = 6/group).

Supplemental Figure 1

a.

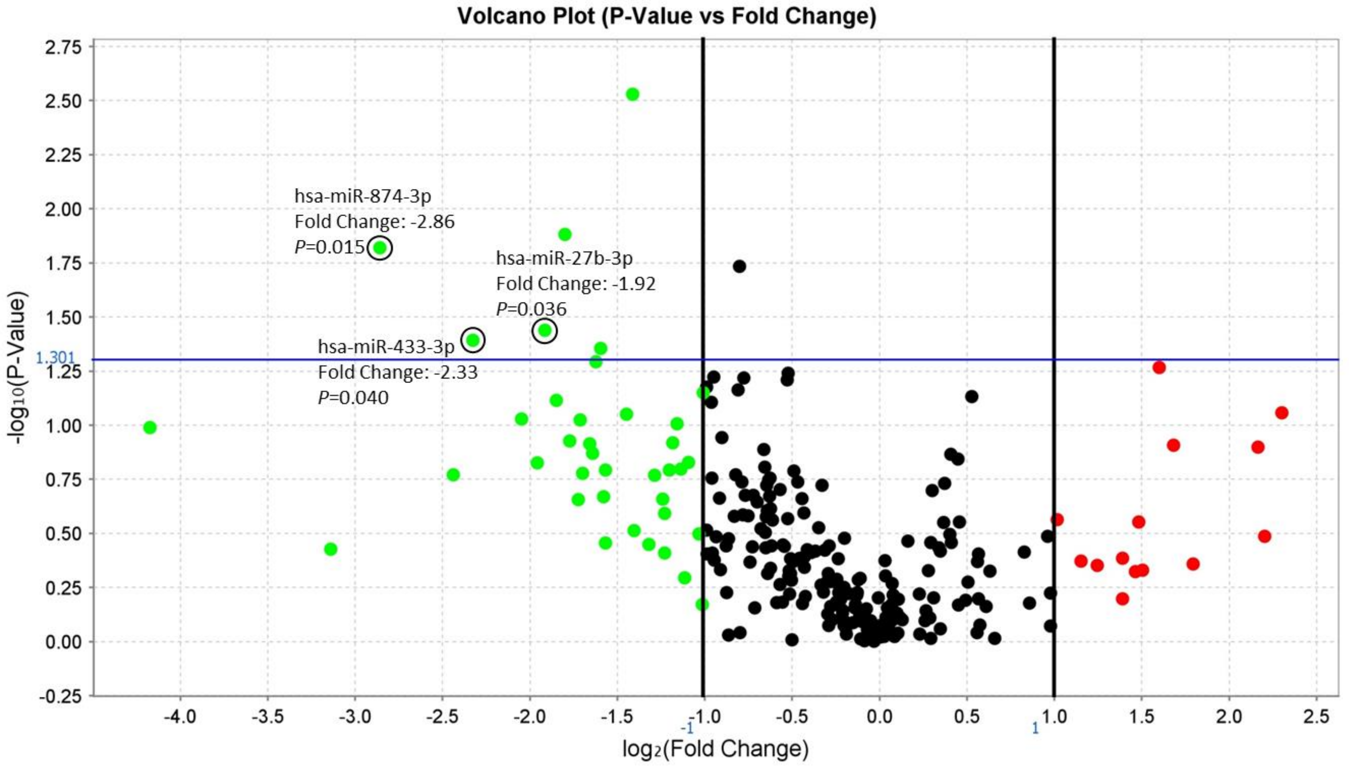

b.

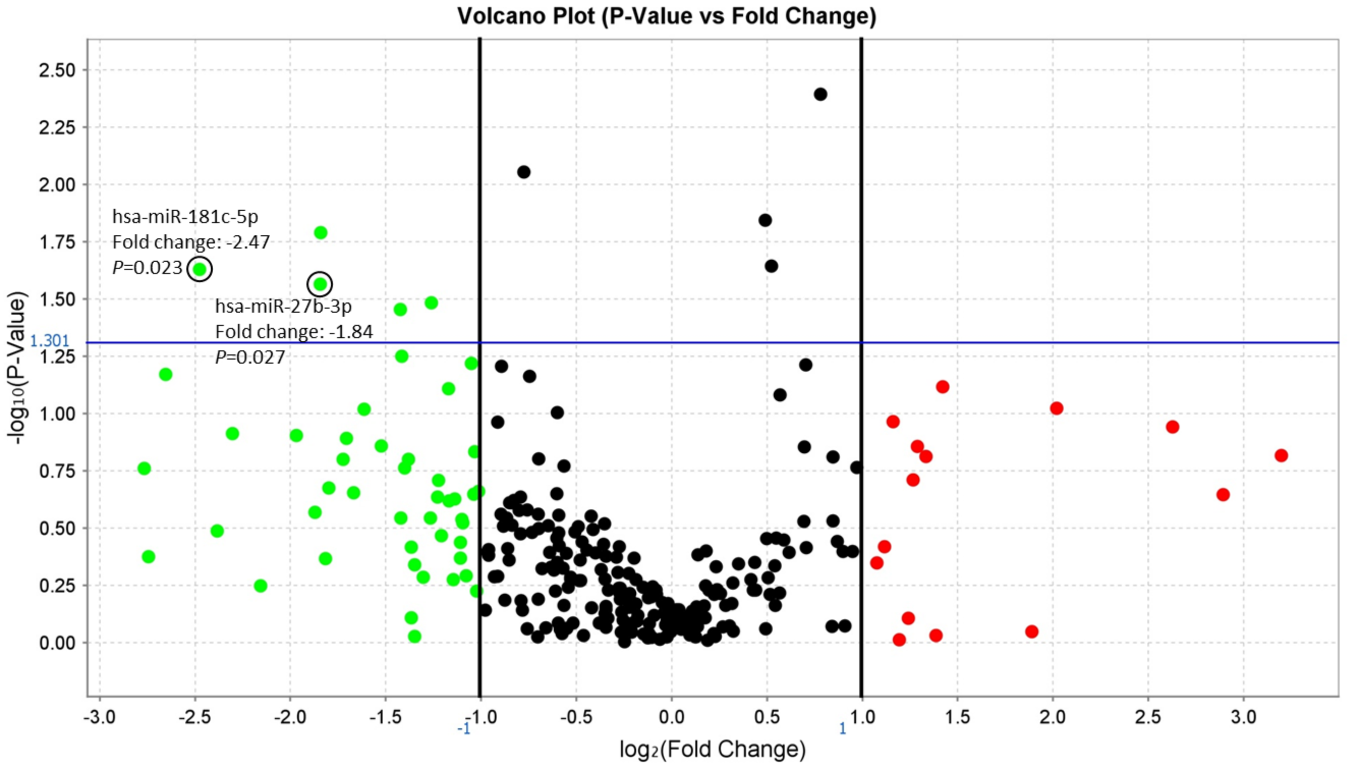

Supplemental Figure 1: Global profiling of miRNAs regulated by rHDL in hypoxia. HCMECs were treated with rHDL (20 μM) for 24 hours prior to hypoxia exposure or stimulation with the inflammatory cytokine TNFα. Expression of 874 miRNAs was assessed using the Megaplex TaqMan Low Density Human miRNA Array. Volcano plots highlighting key miRNAs downregulated (circled) by rHDL when exposed to either (a) hypoxia or (b) TNFα.

Supplemental Figure 2

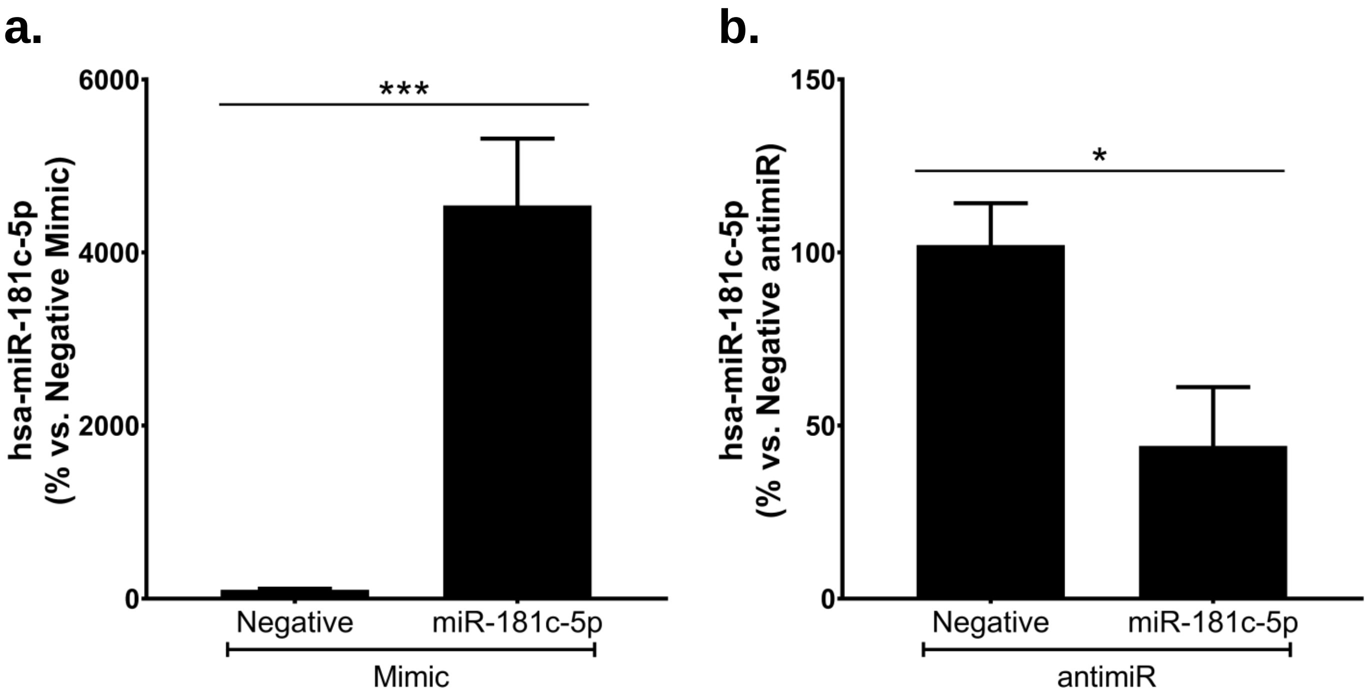

Supplemental Figure 2: hsa-miR-181c-5p levels in human coronary artery endothelial cells transfected with either hsa-miR-181c-5p (a) mimics or (b) antimiRs, with corresponding negative controls. Data expressed as mean±SEM. *P<0.05, ***P<0.001 by t-test.

Supplemental Figure 3

a.

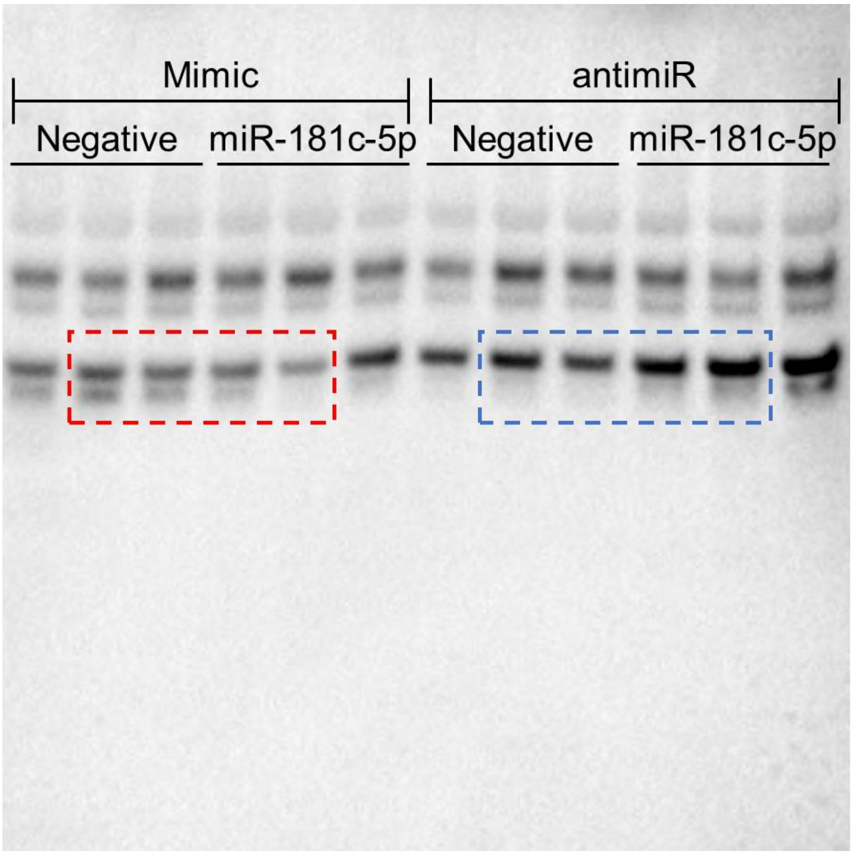

b.

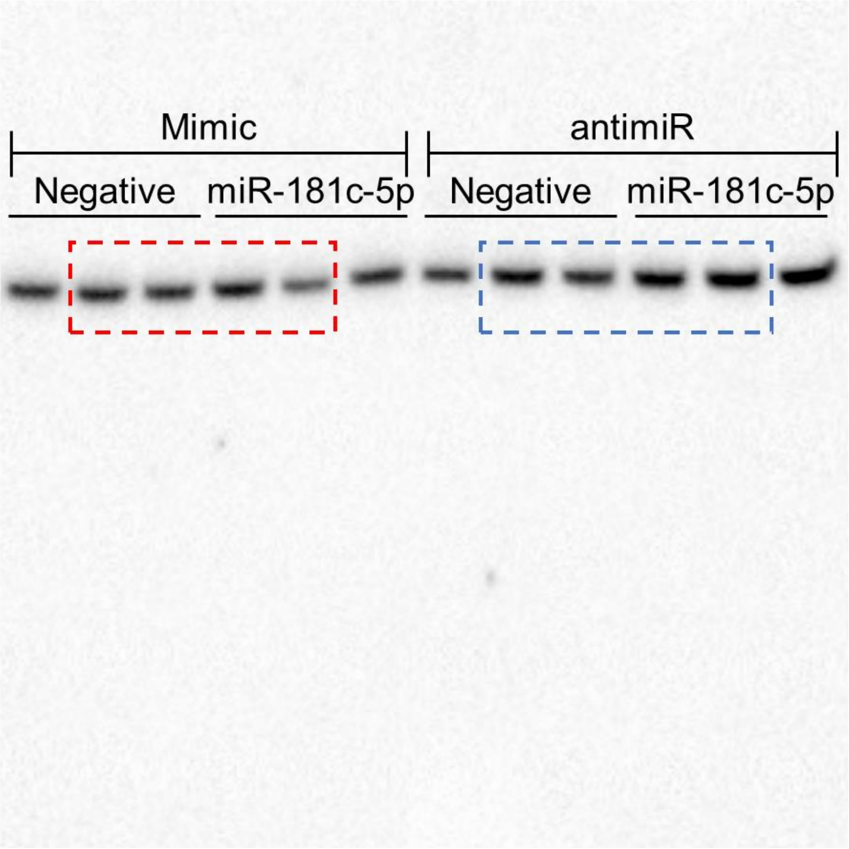

Supplemental Figure 3: Full-length blots of (a) VEGFA and (b) α-tubulin. Red dotted lines show the cropping locations for Negative and hsa-miR-181c-5p Mimics. Blue dotted lines show the cropping locations for the Negative and hsa-miR-181c-5p antimiRs.

Supplemental Figure 4

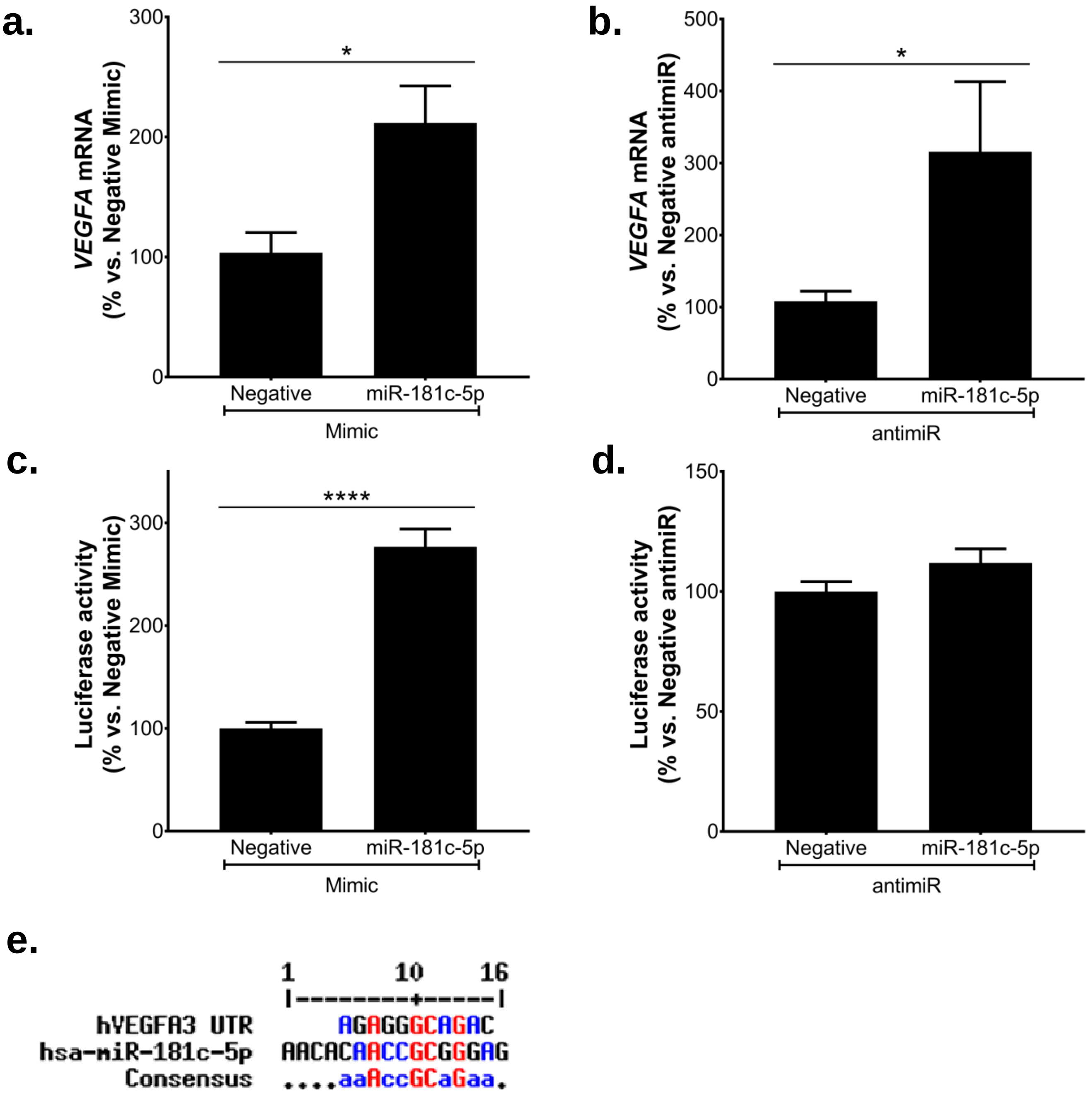

Supplemental Figure 4: *VEGFA* mRNA levels in human coronary artery endothelial cells transfected with either hsa-miR-181c-5p (a) mimics or (b) antimiRs, with corresponding negative controls. Luciferase activity in Ad293T cells co-transfected with VEGFA 3'UTR plasmid and either hsa-miR-181c-5p (c) mimics or (d) antimiRs, with corresponding negative controls. (e) Sequence alignment of human 3'UTR region of *VEGFA* and hsa-miR-181c-5p. Data expressed as mean±SEM. **P*<0.05, *****P*<0.0001 by t-test.
